# Technology Use During the COVID-19 Pandemic and the Ways in Which Technology Can Support Adolescent Well-being: Qualitative Exploratory Study

**DOI:** 10.2196/41694

**Published:** 2023-03-08

**Authors:** Sarah E Rimel, Dina Bam, Laura Farren, Ayana Thaanum, Alessandro Smith, Susanna Y Park, Debra L Boeldt, Chloe A Nicksic Sigmon

**Affiliations:** 1 National Mental Health Innovation Center University of Colorado Anschutz Medical Campus Aurora, CO United States

**Keywords:** adolescents, mental health, technology use, well-being, COVID-19, compassion, social connectedness, mobile phone

## Abstract

**Background:**

Most adolescents in the United States engage with technology. Social isolation and disruptions in activities owing to the COVID-19 pandemic have been linked to worsening mood and overall decreased well-being in adolescents. Although studies on the direct impacts of technology on adolescent well-being and mental health are inconclusive, there are both positive and negative associations depending on various factors, such as how the technology is used and by whom under certain settings.

**Objective:**

This study applied a strengths-based approach and focused on the potential to leverage technology to benefit adolescent well-being during a public health emergency. This study aimed to gain an initial and nuanced understanding of how adolescents have used technology to support their wellness throughout the pandemic. In addition, this study aimed to further motivate future large-scale research on how technology can be leveraged to benefit adolescent well-being.

**Methods:**

This study used an exploratory qualitative approach and was conducted in 2 phases. Phase 1 consisted of interviewing subject matter experts who work with adolescents to inform the creation of a semistructured interview for phase 2. Subject matter experts were recruited from existing connections with the Hemera Foundation and National Mental Health Innovation Center’s (NMHIC) networks. In phase 2, adolescents (aged 14-18 years) were recruited nationally through social media (eg, Facebook, Twitter, LinkedIn, and Instagram) and via email to institutions (eg, high schools, hospitals, and health technology companies). High school and early college interns at NMHIC led the interviews via Zoom (Zoom Video Communications) with an NMHIC staff member on the call in an observational role. A total of 50 adolescents completed interviews regarding their technology use and its role during the COVID-19 pandemic.

**Results:**

The overarching themes identified from the data were COVID-19’s impact on adolescent lives, positive role of technology, negative role of technology, and resiliency. Adolescents engaged with technology as a way to foster and maintain connection in a time of extended isolation. However, they also demonstrated an awareness of when technology was negatively affecting their well-being, prompting them to turn to other fulfilling activities that do not involve technology.

**Conclusions:**

This study highlights how adolescents have used technology for well-being throughout the COVID-19 pandemic. Guidelines based on insights from the results of this study were created for adolescents, parents, caregivers, and teachers to provide recommendations for how adolescents can use technology to bolster overall well-being. Adolescents’ ability to recognize when they need to engage in nontechnology-related activities and their ability to use technology to reach a larger community indicate that technology can be facilitated in positive ways to benefit their overall well-being. Future research should focus on increasing the generalizability of recommendations and identifying additional ways to leverage mental health technologies.

## Introduction

### Background

Empirical evidence supports a pervasive, negative impact of previous outbreaks or epidemics on the population’s mental health, particularly when the outbreaks require quarantine [1,2]. The COVID-19 pandemic has negatively affected the well-being of adolescents across the country, affecting their ability to access support, confidence, and resources to thrive [3]. Research indicates that public health interventions such as social distancing and quarantine have had prolonged negative effects on individuals’ mental health, including increased levels of depression and anxiety, loneliness, and self-harm [4]. The importance of this impact is emphasized by the national emergency in children’s mental health declared by the American Academy of Pediatrics, American Academy of Child and Adolescent Psychiatry, and Children’s Hospital Association [5,6]. According to the National Survey of Children’s Health, between 2016 and 2020, adolescents’ anxiety and depressive symptoms increased substantially, and following the onset of the COVID-19 pandemic, there were increased diagnoses of adolescent behavioral and conduct issues. Unequivocally, the pandemic and social isolation have had a negative and ongoing impact on mental health and well-being for many, including adolescents. Thus, it is imperative to understand how adolescents have responded to the present public health emergency spurred by COVID-19 and to identify adaptive methods within this population for coping and maintaining well-being [7].

### Prior Research

Technology’s causal relationship with adolescent mental health is inconclusive, but positive and negative associations between technology use and adolescent mental health have been observed. Given that 95% of American adolescents aged between 13 and 17 years report having access to a smartphone and report using their phones to pass the time, connect with people, and learn new things [8,9], there is great potential to leverage technology. However, widespread use of social media and prolonged screen time have raised concerns regarding the mental well-being of adolescents. Although studies do not show a direct causal relationship between depression and social media use, adolescent social media use is associated with depressive and anxiety symptoms, loss of sleep, and loss of self-esteem [10,11]. Furthermore, social media use exposes adolescents to images and messages that contribute to body image issues and eating disorders [12].

In contrast, a large-scale study with >350,000 adolescents found that technology use did not substantially decrease well-being and contributes to <0.5% of overall adolescent well-being [13]. Similar findings of a minimal relationship between technology and decreased well-being were obtained across 2 other studies investigating the amount of screen time and engagement with social media [14]. Although parental concerns regarding adolescent mental health and technology use are warranted, technology can be leveraged to benefit adolescent well-being. According to research conducted by Hopelab and Common Sense Media, adolescents view social media as a crucial resource for remaining connected to others, especially during the COVID-19 pandemic [15]. Another study examining parental and adolescent technology use during the pandemic found that social support and information seeking were the most common uses for social media [16]. It is important to note that the term *technology* encapsulates various types of ever-changing digital tools that ebb and flow in popularity among adolescents.

There are various ways in which adolescents can engage with technology, whether it be passively scrolling through social media posts, actively engaging in digital therapeutic sessions, or posting on websites and chatting with friends. Today, tablets and smartphones are among the most popular digital tools used by adolescents and serve as go-to devices for communication, social media use, gaming, and entertainment, namely, for music and video streaming [17]. For adolescents or digital natives (ie, individuals who have grown up with technology), digital tools such as texting and video chatting serve as an essential resource for emotional support and help seeking. According to Colasante et al [18], digital tools offer immense unique benefits to adolescents seeking emotional support because technology allows individuals to immediately connect with others regardless of time or place. This research aims to focus on the strengths and potential for technology to benefit overall well-being of adolescents, while acknowledging that technology has also been linked to less positive outcomes, and how the technology use is facilitated (eg, parental controls, duration of use, type of content accessed, and existing mental health disorders) can play a large role in how well digital tools facilitate adolescent well-being [18]. Technology can provide more timely and easily accessible support to adolescents learning how to cope with negative emotions [18]. Indeed, evidence also suggests a positive link between technology use and relationship enhancement for adolescents [19]. Finally, it is also worth noting that extensive research supports the effectiveness of various technology platforms (eg, apps and virtual reality) to alleviate stress, depression, anxiety, and other important mental health issues in addition to general wellness support [20-22]. For example, Calear et al [23] found that use of a web-based, self-directed cognitive behavioral therapy program substantially reduced levels of adolescents’ anxiety when compared with the control group. Another study by Whittaker et al [24] found similar results, noting that 82.4% of adolescents found a cognitive behavioral therapy mobile app helpful in reducing negative thoughts, solving problems, and dealing with issues at home and school.

### Technology During the Pandemic

Although there have been spikes in distress among adolescents, technology use and availability of various technology apps and platforms have also increased in recent years. As a result of COVID-19–related restrictions and isolation, adolescents across the country relied on technology more often and in new ways to maintain engagement in the central aspects of their lives and for the continuity of relationships [25]. Although we know that technology is a key component for coping, some information remains largely unknown. For example, the most common and helpful ways adolescents used technology during the onset of the pandemic and how adolescents continued to use technology to maintain positive and compassionate connections with others as the pandemic wore on remains largely unknown. Given the immense potential of technology to support well-being and connection, it is imperative to understand the details of how technology is already being effectively used and how it can be scaled and leveraged more broadly for adolescent well-being.

Using technology-based solutions is essential to lessen the burden on the mental health system and to address the overwhelming demand for quality mental health services. As many individuals have access to technology, if implemented successfully and in a sustainable manner, it can be an effective resource for helping individuals stay connected with peers, build resiliency and coping skills, and seek and offer support.

### This Study

This qualitative study aimed to explore the ways in which adolescents engaged with technology during the COVID-19 pandemic. We used an exploratory qualitative approach to obtain a nuanced view of the specific ways in which adolescents used technology to benefit their well-being. The results of this study offer a glance at the adolescent world. Using the findings from this study, we developed guidelines that other adolescents, parents, caregivers, and teachers may use to explore how technology can be leveraged to maximize adolescent well-being during challenging times.

## Methods

### Design

**Figure 1:**
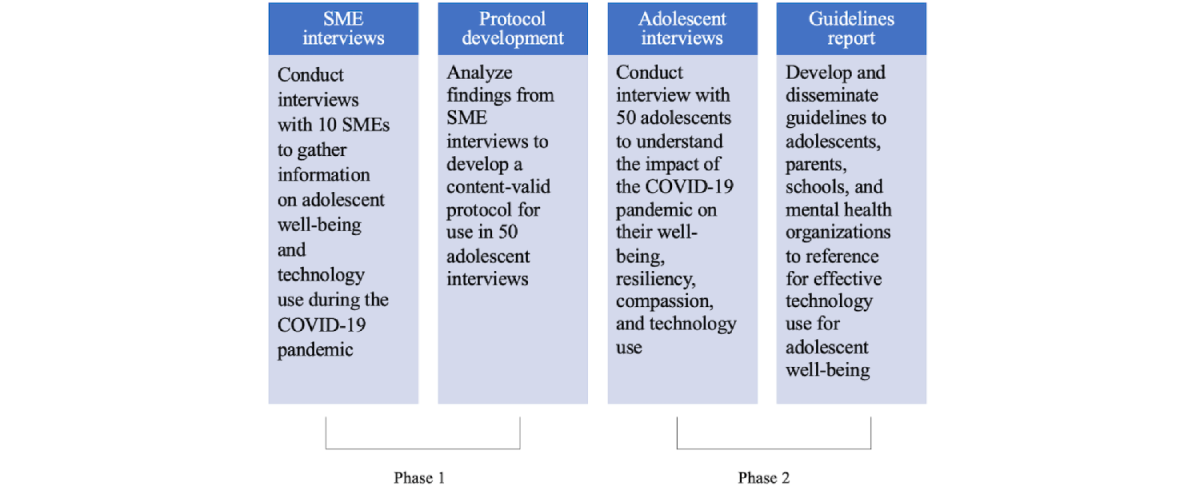
Overall project framework. SME: subject matter expert.

This study consisted of 2 phases (Figure 1). In phase 1, a community-informed research approach was used to engage community members in January 2021. A total of 10 subject matter experts (SMEs), recruited through our network of youth providers and organizations, were interviewed via Zoom. The SMEs represented a wide range of expertise, including mental health providers, teachers, school counselors, and mental health advocates within organizations that work with adolescents. The SME interviews directly informed the study design and subsequent creation of a semistructured interview for adolescents by providing information that would not have been obtained from a literature review alone. For example, the SMEs emphasized that the likelihood of openness and transparency would be increased by interviews led by peers rather than traditional researchers. In addition, based on feedback from SMEs about the differences in social distancing and exposure to COVID-19–related deaths experienced among different demographics, we made substantial efforts to recruit a diverse sample of adolescents. In phase 2, interviews were conducted directly with adolescents to obtain information about how they have used technology since the onset of the pandemic.

### Procedure

We recruited participants nationally through social media (Facebook, Instagram, Twitter, LinkedIn, and Reddit), email campaigns, distribution of flyers, and communication with our SMEs and partnerships that our center has developed with community organizations (eg, school systems). Eligibility criteria required participants to be fluent in English, aged 14-18 years, to have access to Wi-Fi, to not be experiencing active psychosis or suicidality, and to live within the United States. Participants were restricted to the United States given that the pandemic was at different stages of quarantine and restriction worldwide and that technology access and common use may differ substantially based on culture and location. We received >200 emails from individuals who expressed an interest in participating in the interviews. However, 66 individuals were not eligible to participate based on the study criteria; most of them were ineligible because they were based outside the United States. A total of 88 individuals were lost to follow-up. A total of 50 interviews were conducted.

High school and undergraduate research interns conducted interviews with all participants. A research staff member was also on the call to provide support and input as needed. Interns were trained in semistructured interviewing techniques and followed an interview script to conduct the interview (Multimedia Appendix 1 provides a list of the interview questions). They were also trained on how to adhere to the safety protocol should adolescents disclose high-risk behavior. Interviews were scheduled for 60 minutes and were completed via Zoom. Interviewees were by themselves in their homes or at a location of their choice during the interviews, and they were compensated with an electronic gift card for their participation in the study.

### Participants

The participants’ age ranged from 14 to 18 years. Most of the participants (36/50, 72%) identified their sex as female. Furthermore, 66% (33/50) of the participants identified as White, 12% (6/50) as African American or Black, 8% (4/50) as Asian, and 14% (7/50) as other. Of the 50 participants, 8 (16%) indicated Hispanic or Latinx origin. Participants also indicated their sexual orientation, of which 72% (36/50) identified as heterosexual and 18% (9/50) identified as bisexual. Homosexual, queer, and pansexual identities were indicated by 1 participant for each category, and 2 participants preferred not to answer. The sexual orientation of the sample was consistent with national demographic data [26]. Although 54% (27/50) of the sample was from Colorado, there were participants from 12 other states, including New York, Pennsylvania, California, Texas, Vermont, and Florida. Table 1 provides the detailed demographic information.

**Table 1:** Demographic data of participants.

Demographic characteristics		Values, n (%)
**Age (years)**		
	14	12 (24)
	15	6 (12)
	16	9 (18)
	17	11 (22)
	18	12 (24)
**Sex**		
	Female	36 (72)
	Male	14 (28)
**State of residence**		
	Colorado	27 (54)
	California	4 (8)
	New York	3 (6)
	Florida	2 (4)
	Texas	2 (4)
	Arizona	2 (4)
	Oregon	2 (4)
	North Carolina	1 (2)
	Pennsylvania	1 (2)
	Utah	1 (2)
	Vermont	1 (2)
	Virginia	1 (2)
	Washington	1 (2)
	Illinois	1 (2)
	Connecticut	1 (2)
**Residential environment**		
	Urban	42 (84)
	Rural	8 (16)
**Sexual orientation**		
	Heterosexual	36 (72)
	Bisexual	9 (18)
	Homosexual	1 (2)
	Queer	1 (2)
	Pansexual	1 (2)
	Asexual	0 (0)
	Prefer not to answer	2 (4)
**Racial identity**		
	White	33 (66)
	Black or African American	6 (12)
	Asian	4 (8)
	American Indian or Alaska Native	0 (0)
	Native Hawaiian or other Pacific Islander	0 (0)
	Other	7 (14)
**Hispanic or Latinx origin**		
	Not of Hispanic, Latinx, or Spanish origin	42 (84)
	Mexican, Mexican American, or Chicanx	3 (6)
	Cuban	1 (2)
	Puerto Rican	0 (0)
	Another Hispanic, Latinx, or Spanish origin	4 (8)

### Ethics Approval and Informed Consent

The study was approved by the Colorado Multiple Institutional Review Board, the institutional review board for the University of Colorado Anschutz Campus (#21-2801). Participant recruitment began in June 2021 and ended in November 2021. Participants (and their parents, if they were aged <18 years) provided informed consent before completing the interviews. The study data reviewed in this manuscript were deidentified and linked only to the participant ID number.

### Qualitative Interview Analysis

We followed the best practice guidelines for qualitative research in public health to analyze our data [27]. An initial codebook was created by reviewing several interviews and identifying the repeating concepts and themes. The codebook defined 4 initial domains to be used in the analysis: COVID-19, technology, emotional regulation and compassion, and mental health. Double coding was conducted to ensure an intercoder reliability of at least 75% agreement on theme interpretation. After transcription, code queries were run for each of the 4 domains of the established codebook. Commonalities and representative themes across the quotes were analyzed, and 4 overarching themes were produced (outlined in the *Results* section). On the basis of the empirical evidence suggesting differential impact of the pandemic based on demographic factors and feedback from our SMEs in phase 1 that adolescents from marginalized groups may be more isolated or more exposed to pandemic-related death and grief [28], we explored whether the themes on technology use that emerged from our data differed based on key demographics, such as gender identity, sexual orientation, and race. Interviews were transcribed using Otter Transcription Software and coded using NVivo (version 12; QSR International) qualitative software [29].

## Results

### Overview

A total of 4 major themes emerged from our qualitative analysis of all 50 interviews: COVID-19’s impact on adolescent lives, positive role of technology, negative role of technology, and resiliency. We observed that the participants experienced major shifts in their daily lives, requiring them to adjust and navigate their relationships in new ways. As adolescents adjusted to a *new normal*, technology played a consistent role in facilitating relationships and community. Where appropriate, relevant participant demographic data were included. Sex, age, race, Hispanic or Latinx origin, and sexual orientation of the participants were indicated when available.

### Impact of the COVID-19 Pandemic on Adolescent Lives

The COVID-19 pandemic presented adolescents with many new and rapid changes. Where adolescents traditionally would be engaging in school, extracurricular activities, family gatherings, and other social events in person, they were instead forced into web-based settings or could not participate in activities entirely. Unsurprisingly, this led to increased feelings of loneliness, isolation, sadness, and boredom among many adolescents. Social distancing and quarantine mandates sparked rapid changes in daily routines that were both unexpected and unfamiliar. Although some adolescents cited technology as helpful in coping with their new norm, others felt that engaging virtually created additional barriers to connecting with others and maintaining relationships.

Relationships with family and friends changed as adolescents were forced to spend most of their time at home. Structural changes in routine pushed adolescents to find a new routine and adjust the ways in which they engaged with their environment. Social distancing and quarantining created many unexpected and unfamiliar situations for adolescents. Some adolescents found social distancing to be a particularly challenging shift. Being unable to show physical affection toward family members or friends was distressing for some adolescents. This, coupled with the inability to see friends, family, and classmates, led to many adolescents experiencing sadness and boredom. Adolescents discussed distress associated with the inability to engage in activities in ways they usually would. One participant noted the pandemic’s effect on their well-being and how it changed the way they experienced the activities that they loved:

As much as I loved to dance, and I could not go like a week without dancing, I was just like, this is excruciating to be in my bedroom or be in the basement trying to dance.

Female, 18 years, Asian, non-Hispanic or Latinx, heterosexual

Adolescents also reported increased feelings of loneliness and isolation because of the discontinuation of social events, restrictions around gatherings, and the unexpected need to isolate or quarantine. Although technology served as a helpful tool for connection, there was also confusion and anxiety around the severity and how long the pandemic would last. This led to exacerbated feelings of loneliness:

So, I think being even more isolated from the few things that brought a lot of joy and happiness in my life, like activities outside of school, seeing my friends at school just kind of heightened that feeling of isolation, for sure.

Female, 18 years, White, non-Hispanic or Latinx, heterosexual

Resources that were typically accessible through in-person interactions at school became more challenging, as they were often isolated in virtual environments, which were not always conducive to seeking help. Adolescents noted that in a prepandemic world, school counselors or trusted adults in academic settings were easily accessible and served as valuable resources when seeking help:

If schools were open, then I would probably have the opportunity to ask my counselor in person, or even some of my teachers, but since school wasn’t an option...it kind of made things a little bit difficult for me.

Female, 18 years, Hispanic or Latinx, heterosexual

Adolescents described their schools’ response to difficult times during the COVID-19 pandemic. One participant talked about their school hosting a virtual vigil after a mass shooting near campus, which made her feel supported by her school. Others mentioned ways in which their schools prompted connections between students through group chats and class introduction pages on the web. A minority of participants shared that teachers and school administrators were not understanding and that web-based classes were disorganized and confusing, which contributed to distress. Overall, teachers were described as caring:

But teachers are a lot more understanding. Honestly, I was much more aware that they’re nicer with assignments and easier with grading.

Male, 15 years, White, non-Hispanic or Latinx, heterosexual

### Positive Role of Technology

Although in some ways the COVID-19 pandemic contributed to feelings of isolation and increased anxiety levels, many participants noted positive changes in that they could foster deeper connections with their peers over a mutual understanding of the pandemic. Many participants indicated that communicating more clearly and about more complex topics improved their well-being. Peer support was identified as crucial and flourished. Participants also noted spending more time with their family and becoming closer with their family. Family was one of the essential support systems for many participants and was discussed at length, often emphasizing siblings. Friendships also changed owing to the pandemic. Although some adolescents noted negative changes, such as their friend groups growing smaller owing to less frequent interactions or pandemic-related differences of opinions, the relationships that lasted grew much stronger:

Well, with one of my friends, I’ve tried to be more open so she can talk through things with me. We’ve done more activities together and we’ve become closer friends, even though we were apart the entire time during COVID. Because she’s a very closed off person, I was saying, “it’s okay, you can open up” and so she started telling me things and that definitely got us closer.

Female, 14 years, Asian, non-Hispanic or Latinx, heterosexual

Technology served as a resource in fostering well-being through its ability to bolster connections and compassion for peers, friends, and family members. Adolescents noted their concern for their friends’ mental health and took the initiative to engage in vulnerable conversations:

I would say it honestly has made vulnerability a lot easier. I think some people did get to really low places where they had to turn to people for support, and I think that helped a lot of people realize that that’s an okay thing to do and that it does help. And also, we all have recognized that even if we’re just texting for three hours, it’s still some sort of a connection.

Female, 18 years, White, non-Hispanic or Latinx, gay or lesbian

Adolescents used a variety of software and hardware throughout the pandemic. Some applications facilitated communication with friends and family, whereas schools and teachers relied heavily on technology to educate students through distance learning. Some adolescents mentioned using technology for sleep aids and mental health apps as well.

Technology also facilitated communication with larger audiences beyond adolescents’ local communities. Although the world was “on pause” during the pandemic, technology allowed adolescents to stay connected and engage with the people, social causes, and activities that are most important to them. Participants reported playing video games via Xbox (Microsoft Corp) and PlayStation (Sony Interactive Entertainment), sharing stories on TikTok and Discord, and exchanging artwork through various social media platforms with adolescents who they would not have otherwise had the chance to meet.

Technology opened the door for adolescents to pursue mental health care, which they had not previously felt comfortable doing. Technology allowed direct access to providers and care by reducing several barriers to traditional care, such as time, travel, cost, and convenience:

I had a therapist during COVID too, which was the first time I’ve done therapy. So, I feel like I overcame a lot of mental barriers during that time that I didn’t really know I had but I was able to work through in COVID with my therapist. I mean, I did it virtually so that was very helpful.

Female, 18 years, African American and White, Hispanic or Latinx, heterosexual

Not only was mental health and wellness a prominent topic of adolescents’ web-based discussions during the COVID-19 pandemic, but celebrities and other public figures also used social media to share their own experiences. They also provided information on tips for maintaining well-being and seeking care for mental health. This shift helped to reduce the stigma surrounding mental health and care seeking:

So I watch a lot of TikToks and everything, and just people sharing their stories on that and hearing what other people have to say, knowing that I’m not alone in what I’m feeling has really helped me to not feel isolated. And once I started sharing more, I realized that it actually helps me a lot and isolating myself makes the situation worse.

Female, 16 years, African American and White, non-Hispanic or Latinx, heterosexual

Technology helped several participants engage in community activities and connect with people that they would not typically encounter in the *real world*. Participants engaged in online support groups and club meetings. Technology also created new opportunities for maintaining involvement with tasks typically performed in person before the pandemic. Several adolescents mentioned that the transition to remote learning and video calls made the college application process more manageable:

I feel like it’s adapted to the fact that we could never go to school again, and we would be able to learn. People could never go to work again, and they would still be able to do their jobs. It has also helped connect people a lot more, like I’ve done college tours online, I don’t have to fly out there, and I can still talk to admissions people because we have Zoom.

Female, 17 years, White, non-Hispanic or Latinx, heterosexual

### Negative Role of Technology

Some participants described technology as “draining” and “exhausting,” whereas others blamed excessive technology use for procrastination and loss of sleep. Adolescents seemed aware that they feel “addicted” to technology and expressed a desire for other tools they could use for mood regulation. Participants described extreme technology fatigue owing to the shift from in-person schooling to remote learning during the COVID-19 pandemic. Many adolescents reported spending ≥5 hours engaging with technology daily and experienced exhaustion and frustration because of these extended periods of technology use. Several participants mentioned that taking breaks from technology by going outside was necessary. One participant stated the following:

I would do anything to get away from it for a couple of minutes.

Female, 18 years, White, non-Hispanic or Latinx, gay or lesbian

Although technology allowed for new ways of engaging with their community, interviewees found value in physical activities outside of technology that brought normalcy to their lives and helped improve or maintain their well-being:

Certainly, the cross-country community helped me out because they’ll push me to stay active. Because I know for sure that if I was inactive, I would feel a lot worse. When I don’t exercise or something like that, my brain doesn’t work as well.

Male, 16 years, White, non-Hispanic or Latinx, heterosexual

Most smartphones allow for “screen-time” tracking, which shows users how much time they spend on their phones across different platforms. Many adolescents mentioned wanting to reduce their technology use after seeing data from their screen-time reports that shocked them:

I just don’t like being so reliant on my phone all the time. I get anxious when it’s not near me all the time too. I never used to be like that and so I’m kind of hoping to ease off with my phone more.

Female, 16 years, African American and White, non-Hispanic or Latinx, heterosexual

Participants also experienced periods of self-doubt and self-criticism because of making comparisons when engaging with social media. Adolescents noted the presence of influencers in their lives as setting unrealistic expectations and standards causing “shame and feeling self-conscious.” They also mentioned feeling frustrated with their peers and celebrities who posted pictures and videos of themselves on the web doing activities during the COVID-19 pandemic while not complying with the Centers for Disease Control and Prevention’s recommendations of wearing a mask, maintaining social distancing, or vaccinating:

I feel like with social media and once people started to interact with people in person more, you’re kind of being either left out of things because you lost communication over COVID, feeling as if you’re doing the wrong thing based on body image or what you’re interested in, feeling as if you are not good enough in comparison to others, or that you just want to kind of fit in and be with everybody else instead of being an individual, being outside of the box.

Female, 17 years, African American, non-Hispanic or Latinx, heterosexual

### Resiliency

Resiliency in adolescents is defined by the ability to overcome the negative effects of risk exposure [30]. Despite the difficult situations and experiences that the pandemic commonly placed on individuals, many adolescents exhibited substantial resiliency and provided critical insights into factors that helped them build their resiliency. Many adolescents highlighted that the pandemic provided time for individual reflection and introspection. They noted that although they experienced some loneliness and isolation, individuals used their time to practice self-care, evaluate their understanding of the world and their communities, and seek help. Individuals reported that using mindfulness apps and e-journals allowed them to reflect on and cope with stressors while increasing their self-awareness to bolster their resiliency:

I think my mental state has been better since I’ve had more time to focus on myself and see where I was at in beginning of the quarantine. So, I learned more about myself and learned about the stuff that I needed to do, and that I needed to focus to become the person that I am now.

Female, 17 years, White, Hispanic or Latinx, heterosexual

Self-care was a common discussion in many interviews. Participants formed new habits and routines during the pandemic to bring more structure to their lives. Adolescents noted that the pandemic provided them with an opportunity to make healthy changes and build their self-confidence:

I feel like with COVID-19, I was able to take a lot more time to focus on my physical image and work on my self-esteem and work on what I want to look like and what I’m trying to work towards.

Female, 17 years, African American, non-Hispanic or Latinx, heterosexual

Many adolescents talked about joining various support groups and seeing therapists and noted the help therapy provided during the pandemic. Many participants noted that seeking peer support or familial support became much easier during the pandemic and that they felt more comfortable being able to ask for help. This was because of an innate understanding among their community of how difficult the pandemic was:

We all checked in on each other a lot. And when someone wasn’t doing well, even if you couldn’t physically be there for them, being able to be like, “okay, step out into your backyard and give me a call,” or like, “I’ll stay up texting you until three in the morning, just making sure that you feel better.” Even if we couldn't have like a physical shoulder to provide for people to cry on, it was still just being able to be there through technology was a really great thing to have.

Female, 18 years, White, non-Hispanic or Latinx, gay or lesbian

Resiliency was further demonstrated among adolescents who took it upon themselves to foster larger communities. Adolescents described various social activism and community involvement opportunities during the COVID-19 pandemic. They engaged in advocacy, including suicide prevention work, youth boards, nonprofit advisory boards, leadership councils, church groups, school clubs, community service activities, and mentorship programs. Technology opened the door for event planning and large-scale movements to transition from the web-based world into the real world:

I’m part of the one Colorado Gender and Sexuality Alliance (GSA) Leadership Council and we were able to share stories from survivors anonymously, and share resources for survivors and information about the Council on Instagram and really gain support that way. We had been using it to share resources and information about all the work we’ve been doing, which is cool because everyone responds to it in such a positive way.

Female, 17 years, White, non-Hispanic or Latinx

The political climate during the pandemic as well as the Black Lives Matter movement prompted adolescents to use technology to share their opinions and educate themselves on social justice matters:

You could tell that we were all isolated and had our own stuff we were going through. So, I felt like the way we used our phones and connected was a big, really big part of how we stay together and connected.

Female, 17 years, White, Hispanic or Latinx, heterosexual

Technology allowed for additional means of activism in that individuals could use technology to participate in causes that are important to them. Adolescents noted that although the pandemic substantially changed their daily activities, participating in activism and social causes in their community contributed to their resiliency and provided meaningful action during times of uncertainty:

I made a short student documentary about the inequalities within COVID-19 and how different marginalized communities are affected. I talked about the black community and the LGBTQIA community and a lot of the communities that are within the United States.

Female, 17 years, African American, non-Hispanic or Latinx, heterosexual

### Data Analysis Based on Key Demographics

Qualitative analysis comparing female-identifying participants with male-identifying participants did not reveal any remarkable differences in their experiences with the COVID-19 pandemic, relationship with technology, mental health, and emotional regulation and compassion. The results were similar when examining participants who identified as heterosexual versus lesbian, gay, bisexual, transgender, queer, and others (LGBTQ+). Consistent with the literature on loneliness and isolation in the LGBTQ+ community during the COVID-19 pandemic, 1 participant (Female, 15 years, White, non-Hispanic or Latinx, bisexual) indicated that the pandemic was pivotal to strengthening their connections to LGBTQ+ people and youth. They also used technology by starting a subreddit to connect with others who have been diagnosed with the same chronic disease with which they have been diagnosed.

Among participants who identified as a person of color, there were discussions of adolescents actively participating in or creating community spaces for other people of color. For example, 1 participant (Female, 17 years, African American, non-Hispanic or Latinx, heterosexual) created a student documentary for marginalized communities, actively participated in diversity calls, and participated in marches and protests. Participants who were already connected to their racial or ethnic communities found the novel ways of staying connected during various waves of the pandemic to be easier. However, for 1 participant (Female, 18 years, Asian, non-Hispanic or Latinx, heterosexual) engaging with technology in novels ways due to the pandemic did not equate to less feelings of loneliness or isolation.

### Guidelines for Technology Use

From the interview data, our team developed guidelines (Multimedia Appendix 2) to provide specific recommendations for fostering well-being through technology, and these guidelines are intended to be disseminated as a resource for parents, teachers, caregivers, and adolescents themselves. These recommendations directly address the pervasive feelings of loneliness and isolation commonly discussed among the interview participants. In addition, the guidelines offer directions for effective social media use, taking breaks from technology to curb technology fatigue, reflecting on individual experiences by using mood tracking and e-journal applications, and using technology to seek help. More specifically, these guidelines provide mental health resources that can help alleviate mental health issues among this population.

## Discussion

### Principal Findings

This study provided insight into how adolescents have leveraged technologies such as mobile apps, web-based platforms, and social media during the COVID-19 pandemic to achieve connection and well-being. We found that despite all the unexpected changes, anxiety, and grief resulting from the pandemic, adolescents found ways to cope and that technology was a critical component of many coping strategies.

Adolescents reported that their typical avenues for help seeking were blocked but noted that access to trusted professionals became easier in many ways because of the options for web-based therapy. Adolescents also reported using technology to maintain and even increase their well-being. Our findings are consistent with the literature in that individuals found that connecting with peers was critical for their well-being [31]. Participants reported that they relied on technology to create new avenues for connecting with family and peers. They also reported that using social media not only to communicate but also as a platform for comedic relief (ie, watching humorous videos) helped increase levels of happiness. In addition, adolescents reported that technology was critical for allowing them to continue school and stated that it enabled them to participate in social activism and community efforts consistent with their values. Participants also noted that technology served as an information resource and was a helpful tool in reaching out to others who they saw struggling during the pandemic.

Although the interviews yielded information consistent with the known drawbacks of technology, such as technology fatigue and the ability to create unhelpful comparisons, adolescents reported knowledge and skills for coping with or avoiding the downsides, such as taking technology breaks and blocking social media accounts that facilitate comparisons. Overall, our findings overwhelmingly highlighted the unique and essential support technology provided for adolescents’ resiliency and well-being during the pandemic. The interview data also offer insight into how technology can effectively foster well-being in the long term and how it can be leveraged after the pandemic. As such, we developed guidelines for adolescent technology use based directly on our findings and are intended to provide practical guidance to help adolescents avoid common pitfalls and maximize the benefits that technology has to offer.

### Limitations

Although the SME interviews provided invaluable insight and direction for the development of the adolescent interview protocol and script, there were limitations to this process and our study design. One important limitation was the sample size of only 50 adolescents and the strict eligibility criteria used to conduct the study activities safely in a virtual environment. Adolescents who did not have access to Wi-Fi at the time of the study, were not fluent in English, or who may have been experiencing active psychosis or active suicidality were excluded from the study. The study sample was also not representative with regard to gender or racial identity. These criteria make it more difficult to generalize the findings. In addition, because there were no objective or quantitative measures included in the data collection to assess participant well-being, depression, or anxiety, it is difficult to draw conclusions about which adolescents our guidelines will help the most. We also must acknowledge the potential bias of this sample; as recruitment was conducted through posts on social media and other platforms, study participants were adolescents already engaged with technology, and therefore, it is difficult to analyze how these findings or recommendations might apply to adolescents not on social media or who use technology less frequently. This study was conducted with the idea that it represents an initial step in the process of understanding adolescent technology use and ways in which technology can benefit well-being. Future research is necessary for more broad-based generalization, and it should be built upon the findings of this study by directly addressing and minimizing the stated limitations.

### Future Directions

Moving forward, it is vital to deepen our understanding of how adolescents have responded to the disruptions of the COVID-19 pandemic and how technology can be used to enhance their well-being, particularly during times of stress. Our study offers a first step into exploring the benefits of adolescent technology use. With the aim of increasing generalizability of the findings, future research should expand eligibility criteria to include non-English speaking or non-US residents and should collect both quantitative and qualitative data related to outcomes from a larger sample of adolescents. To better understand the limitations of recommendations, future research should also dive deeper into the lives of institutionally marginalized adolescents, including adolescents with less access to technology and less time or fewer resources to devote to prioritizing well-being. Using a longitudinal research design with a larger and more demographically representative sample would also maximize generalizability and help to decipher which recommendations might be most helpful. From a development and implementation perspective, it is essential that future studies examine the feasibility, acceptability, and effectiveness of specific digital health tools used in both clinical and nonclinical settings in the mental health care system. These efforts will help identify solutions that will effectively and substantially reduce the demand on the system, which the pandemic has only exacerbated [32]. Overall, research aiming to better understand key mechanisms to foster adolescent resiliency and coping is essential for the well-being of this and future generations.

## Appendix

Multimedia Appendix 1 Semistructured interview questions.

Multimedia Appendix 2 Teens and technology guidelines.

## Abbreviations

LGBTQ+: lesbian, gay, bisexual, transgender, queer, and others
NMHIC: National Mental Health Innovation Center
SME: subject matter expert

## Data Availability

The data generated from this study are available from the corresponding author on reasonable request.
