# Supplementary material for: Technology Use During the COVID-19 Pandemic and the Ways in Which Technology Can Support Adolescent Well-being: Qualitative Exploratory Study
Source: JMIR Form Res. 2023 Mar 8;7:e41694. doi: 10.2196/41694 (PMC9997705; doi:10.2196/41694)
Supplement: Multimedia Appendix 1 [file formative_v7i1e41694_app1.pdf]

Technology Use During COVID-19: Semi Structured Interview Questions

1. We know 2020 has been a rollercoaster for a lot of people. If you were to meet someone who didn't know about COVID-19 or the year 2020, what would you tell them about your personal experience?
    a. PROMPT: Tell me about a specific story or memory that you can recall that really highlights the experience you just shared.

2. We know that many adolescents have had both positive and negative emotional experiences during the past year due to COVID-19. Today we want to understand what things have helped you throughout the pandemic. What have been some things you've done to help you throughout the pandemic?
    a. As the pandemic has continued, how has your wellbeing changed (I.e., gotten better, worse, stayed the same)?
        i. When we say wellbeing, we mean the "state of being comfortable, safe, healthy, or happy."
    b. In what ways have you addressed any challenges to your mental health and wellbeing during COVID?
        i. Responses to difficult questions:
            1. "It sounds like that was really hard for you. Thank you for sharing."
            2. "Your insights are helpful to us. Thank you for your openness."
            3. "We have heard many people say they are feeling that way. Thank you for sharing."

3. In general, in what ways have you engaged with technology to help maintain or improve your wellbeing?
    a. Remember: Wellbeing meaning the "state of being comfortable, safe, healthy, or happy"

4. List the tech you've been using from what you use most to least.
    a. PROMPT: Here we mean any hardware, like gaming systems or VR, as well as software, like Snapchat and Zoom.
    b. OR What are the top 3-5 technologies or apps that you use regularly?

5. In what ways has technology played a role in your life since the onset of COVID?
    a. PROMPT: Can you tell me about a specific memory related to what you just said?

6. In what ways has your technology use changed since the onset of COVID?
    a. For example, have you been using new technology? Different technology? Certain technologies more than others?
    b. How involved have your parents/caregivers been in helping you find new tech or learn new tech use? Do you have any restrictions or rules around tech use?

7. How have you seen your friends and family affected by the pandemic?

8. What are some activities you've engaged in in order to help peers, friends, family, or those in your community since COVID began?
   a. What responses have you received from these individuals?
   b. How has that helped your wellbeing?

9. We've been learning that people's communities are playing a big part in how they are experiencing the pandemic. How would you describe your community?

   a. PROMPT: By community, we mean friend groups, peers at school, neighborhoods, families, teammates, religious groups, online networks, video gaming groups, political or advocacy groups, or any other formal or informal way people feel connected or identify with others.
   b. In what ways has your community been impacted by the pandemic?
      i. What are some ways your community has remained connected throughout COVID?

10. Can you tell us about the ways you've used tech platforms to offer support others in your community?

   a. How is this different during COVID compared to what it would have looked like before?
   b. How might it change once the pandemic is over?

11. Tell us about some of the activities besides school you've engaged in since COVID began?
   a. More explicitly, how have these activities helped you with your resilience or your ability to quickly recover from stressful events or situations?
   b. How have these activities intensified the stressors or made you feel worse?

12. Since the onset of COVID, can you tell us any new ways that you've used technology to remain connected with activities outside of school? (e.g., extra-curricular groups, volunteer groups, sports/exercising)
   a. (If they do): in what ways do the tech platforms that you use make you feel supported or empowered?
   b. How do you see tech helping others to feel this way?

13. Many people have felt lonely during the pandemic. How has technology helped you address loneliness or isolation?

   a. Are there any ways in which technology has contributed to feelings of more loneliness? If so, can you share your thoughts?

14. We've heard a lot about tech fatigue in the last year, in what ways have you felt tech fatigue, if at
all?

   a. PROMPT: When we say tech fatigue, we mean feeling worn out or tired due to excessive time on digital platforms. Also, the urge to avoid tech, anxiety when using tech that wasn't there before, feeling more stressed in general and less motivated that might be related to the time spent on digital platforms.

b. What are some things you’ve done to overcome the fatigue you’ve felt from so much daily tech use?
    i. Can you provide a specific example from what you just shared?
        1. How well do you feel these things have help with tech fatigue?

15. How have you been using technology in school?
    a. Describe how it has changed since the COVID pandemic began?
    b. What’s been effective about those changes?
    c. How have your parents/caregivers been involved in helping you manage school and technology related to school?
    d. Has your school been responsive to tech fatigue? If so, describe what they’ve done to help reduce that fatigue.
        i. Tell us an example of how this has or has not helped with your overall wellbeing?
