# Supplementary material for: Technology Use During the COVID-19 Pandemic and the Ways in Which Technology Can Support Adolescent Well-being: Qualitative Exploratory Study
Source: JMIR Form Res. 2023 Mar 8;7:e41694. doi: 10.2196/41694 (PMC9997705; doi:10.2196/41694)
Supplement: Multimedia Appendix 2 [file formative_v7i1e41694_app2.pdf]

TEENS AND TECHNOLOGY GUIDELINES

The following guidelines were developed as a result of a project in which 50 adolescents were interviewed about their experience with technology during the COVID-19 pandemic. These guidelines can be used to inform adolescents, parents, teachers, and caregivers on how to leverage technology to build resilience, compassion, connection, and support wellbeing.

1 Feeling lonely or isolated?

- Video chat with friends and family using Facetime, Google Duo, Skype, or Zoom.
- Send thoughtful or funny memes to friends on Instagram to feel more connected and boost both of your moods.
- Say hello through a video message service like Marco Polo or Snapchat.
- Share and collaborate with family or friends on Spotify or Apple Music music playlists.

2 Feeling bored?

Explore online platforms (such as YouTube) for various engaging activities, including academic tutorials, DIY projects, cooking, etc. Learn new skills and share your projects with your peers through video chat or social media posts.

3 It is always okay to reach out for help—we all need help at times!

Find out how to reach out to your counselors using your school resource website. Call or text friends or trusted adults when you need some support. Save these numbers in your phone so that your support systems are ready and easily accessed when you need them.

4 Schedule tech breaks.

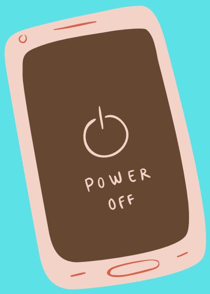

Get some water, go for a walk, stretch. Make a list of things that help you feel relaxed, rejuvenated, or energized. Take these breaks daily, even when it doesn't feel like you need them, because it will help you maintain wellbeing in the long run. You can even set timers during your schoolwork or workday to remind yourself to practice these self-care activities.

5 Reflect on who you are following on social media. How is this influencing your self-image?

Social media is powerful – it can build esteem and connection or work against you. Try following multiple positive accounts such as individuals who inspire you, promote self-care, make you feel empowered, and align with your interests.

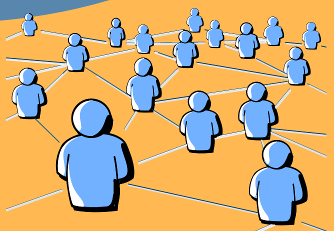

6 Utilize social media or other tech platforms to advocate for what is important to you

Organize meaningful activities (e.g., using social media to raise awareness of mental health stigma or to organize a mural painting or trash clean-up event). When organizing social activities, we recommend remaining active in the algorithm by using hashtags, posting regularly, reposting like-minded posts, and frequently engaging with others. Make your posts interactive using polls, quizzes, and surveys. Define your aesthetic, keep it consistent, create original video content, and follow social activists leading similar efforts.

HEMERA FOUNDATION

National Mental Health Innovation Center
UNIVERSITY OF COLORADO **ANSCHUTZ MEDICAL CAMPUS**

The Avielle Initiative
Building compassion and preventing violence

TEENS AND TECHNOLOGY GUIDELINES

7 Use social media and tech platforms to help others and increase communication.

By advocating about a problem or providing support through technology, your voice can reach others. Many people feel isolated and alone, which sometimes leads to thoughts of hurting themselves, and technology platforms are some people's only means of support. Sharing resources is important.

Resources include: **24/7 National Suicide Prevention Lifeline (1-800-272-TALK (8255)) and the Crisis Text Line (Text TALK to 741-741).**

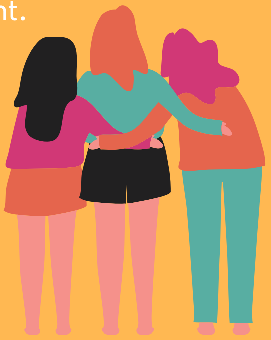

8 Utilize technology for self-expression through positive skill and identity development.

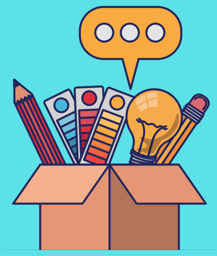

Virtual games such as Minecraft, Let's Create Pottery, Finger Paint Magic, Monument Valley, Rocket League, Animal Crossing, and Roblox offer a space where creativity can flourish, and users can connect and interact with one

9 Create or identify virtual spaces where people can join and be part of the group

(e.g., sub-reddit, group chat via text message, Discord) for things you are interested in or social causes to which you'd like to draw awareness.

10 Use tech to reflect on your daily experiences, track your mood, and use coping skills.

These practices help develop introspection and growth. Although not a substitute for mental health treatment, they can help you cope with stressors (e.g., conflicts with friends or family, virtual learning, etc.) and can increase your own self-awareness. Some e-journals we suggest are: Diaro (free), Day One (free), Happyfeed (free), and Diarium (free). Some mindfulness apps we suggest are: Insight Timer (free), Headspace (subscription), 10% Happier (subscription) and Personal Zen (free).

11 To cope with the unpredictability and ongoing stressors today, seek out factual resources.

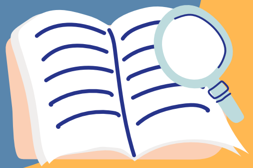

These resources will help you feel confident that you're getting accurate information about safe ways to protect your health.
Resources that may be relevant are:

- CDC (https://www.cdc.gov/)
- WHO (https://www.who.int/)
- DearPandemic (https://dearpandemic.org/)
- Go Ask Alice (https://goaskalice.columbia.edu/)
- Planned Parenthood (https://www.plannedparenthood.org/learn/teens)

12 Work to maintain a sense of normalcy in your schedule and your activities.

Seek virtual solutions to extracurricular activities so that they do not have to be eliminated from your life. Even physical activities (e.g., team sports or lessons) may be done virtually in some capacity; problem solve with adults that lead these activities to see if you can find a solution to keep the activity going.

HEMERA FOUNDATION
National Mental Health Innovation Center
UNIVERSITY OF COLORADO **ANSCHUTZ MEDICAL CAMPUS**
The Avielle Initiative
Building compassion and preventing violence
